# Personality Traits and Physical Activity: Insights from German University Students

**DOI:** 10.3390/ejihpe13080104

**Published:** 2023-08-03

**Authors:** Carsten Müller

**Affiliations:** 1 Wandelwerk, Quality Assurance and Enhancement, FH Münster—University of Applied Sciences, 48149 Münster, Germany; carstenmuellerphd@gmail.com or carsten-mueller@fh-muenster.de or c.mueller@uni-muenster.de; 2 University Sports, University of Münster, 48149 Münster, Germany

**Keywords:** five-factor model, health psychology, exercise, sedentarism, sedentary behavior, resistance training, public health, physical activity guidelines

## Abstract

This study explores the intriguing relationship between personality traits, self-rated fitness (SRF), and physical activity (PA) variables among German university students (N = 4244) and sheds light on the impact of personality on adherence to PA guidelines. Employing an online cross-sectional study, the short-form of the Big Five Inventory-2 assessed five domains of personality traits (Extraversion, Negative Emotionality, Agreeableness, Conscientiousness, and Open-Mindedness). PA, including sitting time, was assessed using the International Physical Activity Questionnaire (short-form). SRF and muscle-strengthening activities (MSA) were assessed with one item each. Multiple linear and logistic regression analyses examined associations of individual personality trait domains and all domains combined with SFR, PA variables, and adherence to PA guidelines, controlling for sociodemographic, behavioral, and (mental) health covariates. Most reliably, Extraversion and Conscientiousness revealed positive associations with PA variables, while Negative Emotionality yielded inverse relationships with PA variables. For instance, each unit increase in Extraversion corresponded to an additional 17 min of weekly MSA. On the contrary, daily sitting time was unrelated to personality. Of note, high Open-Mindedness was associated with lower odds for adhering to current PA guidelines. The findings have implications for developing targeted interventions that promote a physically active lifestyle and support students’ well-being and academic success.

## 1. Introduction

In Germany, the student population in higher education currently stands at approximately 2.9 million, representing the largest status group within universities [1]. Pursuing higher education entails various challenges, such as managing academic responsibilities alongside daily tasks, navigating social adjustments by leaving behind familiar relationships, and assuming personal accountability for health-related behaviors. In addition to maintaining a healthy diet and refraining from substance abuse, one vital aspect of health-promoting behavior is engaging in adequate physical activity (PA).

Extensive evidence consistently demonstrates the undeniable health benefits associated with PA (e.g., [2,3,4]). PA, including exercise training, offers a noninvasive approach to both the prevention and treatment of chronic diseases [5]. Embracing a physically active lifestyle yields primary and secondary preventive effects, effectively reducing the risk of chronic non-communicable diseases such as cardiovascular disease, diabetes, cancer, hypertension, obesity, depression, and osteoporosis, thereby mitigating premature mortality. The magnitude of risk reduction varies depending on the activity measurement method, with estimates ranging from 20% to more than 50% [3,6]. Moreover, emerging research highlights the positive impact of cardiorespiratory fitness and PA on mental health, in addition to their well-established physical health benefits [7,8,9,10].

The World Health Organization (WHO) has provided comprehensive recommendations for PA and sedentary behavior [11]. According to these guidelines, adults are encouraged to engage in 150–300 min per week of moderate-intensity aerobic physical activity (mPA), 75–150 min per week of vigorous PA (vPA), or an equivalent combination of both. Additionally, regular muscle-strengthening activities (MSA) at least two days per week are recommended, given the well-established physical and mental health benefits associated with strength training [12,13,14]. Furthermore, reducing sedentary behavior is advised as it has adverse health effects independent of PA [15,16,17]. However, the precise dose–response relationship between sedentary behavior and health outcomes remains to be fully elucidated. Nonetheless, recent meta-analyses suggest a progressive increase in the risk of disease and mortality with daily sitting time (ST) exceeding 6 to 8 h [15,18].

Despite the overwhelming evidence highlighting the numerous advantages of maintaining a physically active lifestyle for both physical and mental well-being, a significant proportion of the global population remains insufficiently active. Alarmingly, over 80% of children and adolescents, as well as approximately one-quarter of adults worldwide, fail to meet the recommended levels of PA [19,20]. This concerning trend is also observed among students, as indicated by previous research. Findings suggest that only a minority of students meet the current PA guidelines, while sedentary behavior is prevalent, particularly within the higher education context [21,22,23,24].

The available data strongly suggest the necessity for targeted interventions aimed at promoting PA and exercise and reducing sedentary behaviors among students. In order to develop theory-based approaches for PA promotion, it is crucial to have a comprehensive understanding of the various factors influencing daily PA engagement. These factors can range from broader elements such as policy and regulatory interventions to more specific factors such as individual and interpersonal characteristics [25,26]. Among the personal factors that have garnered significant attention in current research, personality traits play a prominent role. Personality traits are defined as enduring patterns of thoughts, feelings, and behaviors that shape an individual’s unique characteristics and tendencies, known to remain stable over time and consistent across various situations [27]. Understanding the intricate relationship between personality traits and PA can provide insights into individual differences in PA behavior, preferences for specific types of PA, and even the varying effects of behavioral interventions. Such insights hold promise for tailoring interventions and strategies that effectively promote a physically active lifestyle among students.

In recent studies, the ‘Big Five’ personality dimensions have emerged as important factors associated with overall health and, specifically, PA behavior [25,28,29,30,31]. Unlike temporary states, personality traits are relatively stable characteristics that significantly influence an individual’s thoughts, emotions, and actions [32]. The widely accepted ‘Big Five’ model of personality traits encompasses five fundamental dimensions that serve as determinants for lower-order sub-traits, allowing for a more specific and detailed description of individuals [25,31]. These dimensions include the following:

- Extraversion reflects a person’s inclination towards sociability, assertiveness, high energy levels, seeking excitement, and experiencing positive emotions.
- Negative Emotionality pertains to emotional instability, anxiety, self-consciousness, and vulnerability.
- Agreeableness encompasses friendliness, cooperativeness, altruism, trustworthiness, and generosity.
- Conscientiousness exhibits traits such as organization, dutifulness, self-discipline, and a strong orientation towards performance.
- Open-Mindedness (to experience/intellect) refers to being perceptive, creative, reflective, valuing imagination and aesthetics, and having an appreciation for new experiences.

Previous systematic reviews have consistently demonstrated that Extraversion and Conscientiousness exhibit positive correlations, while Negative Emotionality shows a negative correlation with daily PA [33,34]. Open-Mindedness displays a weaker positive correlation, and Agreeableness has not been associated with PA [34]. Preliminary research suggests that the relationship between PA and certain higher-order personality traits, particularly Extraversion, Conscientiousness, and Negative Emotionality, may be dependent on PA intensity, with higher-intensity activities yielding stronger effects [34,35]. Another meta-analysis indicated that personality traits, such as higher Negative Emotionality and lower Conscientiousness, are linked to an increased risk of physical inactivity and higher levels of sedentary behavior [36]. However, the literature specifically examining the association between personality and PA among university students remains limited. In one of the few existing studies among 499 physical education students, associations between PA and Extraversion, Conscientiousness, and Negative Emotionality were confirmed [37]. Despite the relevance of the existing findings for health reports and their potential applicability in higher-education health promotion programs, there is a noticeable dearth of studies investigating the relationship between higher-order personality traits and adherence to PA guidelines in general. Moreover, there remains a significant need for additional research that either confirms or challenges the previous findings, particularly in representative samples of university students encompassing diverse academic disciplines. Such comprehensive data are essential for the development of targeted strategies and interventions that effectively support students’ health behaviors and promote their overall well-being and academic success.

Therefore, the primary objectives of this study were to explore the associations between personality traits and self-rated fitness (SRF), various PA variables including MSA and ST, as well as adherence to PA guidelines, while accounting for the influence of sociodemographic, behavioral, and (mental) health-related covariates among university students. Previous research informed the formulation of four hypotheses for this study:

1. SRF and PA would show positive correlations with Extraversion, Conscientiousness, and Open-Mindedness, and negative correlations with Negative Emotionality. ST would negatively correlate with Conscientiousness and positively with Negative Emotionality, but with overall weak associations.
2. Associations between personality and outcome variables would persist after controlling for covariates.
3. The intensity of aerobic PA would influence the relationship between personality and outcome variables, with higher activity intensity demonstrating stronger associations.
4. Adherence to PA guidelines (combining aerobic PA, MSA, and ST) would be positively associated with Conscientiousness and inversely associated with Negative Emotionality, while the associations with the other personality traits were unclear.

## 2. Materials and Methods

### 2.1. Study Design and Setting

This is an online cross-sectional study conducted in a convenience sample at the University of Münster, Germany. The University of Münster stands as the fifth-largest university in Germany, housing 15 faculties and 21 departments. The various disciplines include social sciences (e.g., psychology, sociology, and political science), natural sciences (e.g., physics, chemistry, and biology), and humanities (e.g., religion, philosophy, and linguistics). Within the student population of around 44,500, females make up approximately 56% of the cohort, while foreign students constitute approximately 7% of the total.

The study adhered to the Checklist for Reporting Results of Internet E-Surveys (CHERRIES) to enhance survey quality [38]. The University Institutional Review Committee (2019-07-TU) granted ethical approval. The study included all regular university students, excluding cross-registered students, auditing students, and senior citizens. A total of 42,630 students received email invitations to participate. The questionnaire consisted of 172 items spread across 15 pages and was available in both German and English to allow the participation of international students. Each student received a unique transaction number along with the invitation email, which provided details on the estimated survey duration (20 to 30 min), voluntary participation, anonymity, data protection, and incentives (e.g., a chance to win VIP tickets for sporting events). Non-respondents received two reminders within a two-week period. Prior to continuing with the study, all students were required to provide mandatory informed consent. The survey was administered using evasys version 8.0 with adaptive questioning (evasys GmbH, Lüneburg, Germany), without randomization of items. Participants had the option to modify their answers on previous pages by using the back button. There was no completeness check, but incomplete surveys were also included.

### 2.2. Data Collection

#### 2.2.1. The Assessment of Personality Traits

The assessment of personality traits utilized the abbreviated version (BFI-2-S) of the Big Five Inventory-2, also known as the Five-Factor Model. Extensive research has demonstrated the favorable psychometric properties of the BFI-2-S, which retains high reliability and validity at the domain level in both its English and German versions [39,40]. Notably, it offers the advantage of reduced assessment time and respondent burden. Participants responded to 30 items on a 5-point Likert scale, ranging from strongly disagree (1) to strongly agree (5). After recoding false-keyed items, the BFI-2-S yields scale values for five domains: Extraversion, Negative Emotionality, Agreeableness, Conscientiousness, and Open-Mindedness.

The present study utilized ipsative mean imputation for domains with two or fewer missing items out of a total of six [41]. Notably, one respondent exhibited two missing data points across two domains, while the remaining participants had at most one missing value per domain. The overall occurrence of missing data was less than 0.15% (167 out of 121,740). In this study, the internal consistency demonstrated good to acceptable levels, comparable to previous studies that assessed the psychometric properties of BFI-2-S [39,40]. Specifically, Cronbach’s α coefficients were as follows: Extraversion = 0.76, Negative Emotionality = 0.82, Agreeableness = 0.70, Conscientiousness = 0.76, and Open-Mindedness = 0.72).

#### 2.2.2. The Assessment of Self-Rated Fitness and Physical Activity

The short form of the International Physical Activity Questionnaire (IPAQ-SF) was used to assess students’ aerobic PA levels and total daily ST [42]. The questionnaire assessed the frequency (days per week) and duration (minutes per day) of vPA, mPA, walking, and daily ST in hours over the preceding week. Valid responses required PA durations of at least 10 min but no more than 180 min in each category, allowing the calculation of weekly MET (metabolic equivalent of task) minutes. Multiplying the frequency and duration by a factor of 8 yielded the number of vigorous MET minutes per week, while factors of 4 and 3.3 were used for calculating moderate and walking MET minutes, respectively. Participants reported their average daily ST in hours for the previous week through a single inquiry.

Criterion validity of the IPAQ-SF has been established in adult populations, demonstrating adequacy against accelerometry (Spearman ρ = 0.30, CI_95%_ 0.23 to 0.36) and satisfactory test–retest reliability (ρ = 0.76, CI_95%_ 0.73 to 0.77) [42]. Additionally, when compared to accelerometer counts and mPA to vPA uniaxial and triaxial cut points, the IPAQ-SF exhibits reasonable validity among university students, with Pearson’s *r* ranging from 0.27 to 0.70 [43]. In this study, the internal consistency of the IPAQ-SF was deemed acceptable (Cronbach’s α = 0.74).

A single item inquired MSA in accordance with the IPAQ-SF by asking “How many of the last seven days did you engage in muscle strengthening activities of at least 10 min per session (e.g., strength training using body weight or resistance training with gym equipment)?”. The volume of MSA was calculated by multiplying frequency (number of days per week) by duration (minutes per training session) [44,45]. Additionally, participants’ SRF was evaluated by inquiring, “How would you rate your current physical fitness?” using a 5-point Likert scale ranging from very good (1) to very poor (5). Similar questions have been employed in previous studies to assess SRF (e.g., [46,47]. Cases with missing data for PA variables were considered as complete missing data and were excluded from the statistical analysis (*n* = 80).

#### 2.2.3. The Requirements for Meeting PA Guidelines

Adherence to the aerobic PA guidelines required accumulating a total volume of at least 600 MET minutes per week [29]. To meet the guidelines for MSA, students were required to report engaging in at least two muscle-strengthening sessions per week. Finally, to establish a criterion for daily ST, a threshold of seven or more hours per day was utilized, drawing from a recent meta-analysis that revealed an incremental rise in the risk of non-communicable diseases and mortality beyond this threshold [15]. As current guidelines do not prescribe a specific cutoff for ST, this threshold was adopted as a practical operationalization.

#### 2.2.4. The Assessment of Potential Confounding Variables

To enhance internal validity by accounting for potential confounding factors, a comprehensive set of predetermined sociodemographic, behavioral, and (mental) health-related variables were included in the analysis. Sociodemographic variables encompassed age (in years), gender (dichotomized as male or female), study workload (categorized into seven levels: (1) <10 h per week to (7) ≥60 h per week), and monthly financial resources classified into five levels (EUR 450, EUR 450–699, EUR 700–949, EUR 950–1150, and EUR >1150).

Among the behavioral factors, smoking status was categorized into three levels: non-smoker, occasional smoker, and daily smoker. Alcohol use was assessed using the Alcohol Use Disorders Identification Test (AUDIT-C), consisting of three items rated on a five-point Likert scale [48,49]. Fruit and vegetable consumption was classified into four levels based on servings per day: (1) 0 servings, (2) 1–2 servings, (3) 3–4 servings, and (4) ≥5 servings. Sleep quality was evaluated using the short Pittsburgh Sleep Quality Index (sPSQI), which involved rating 13 items with time specifications and four-point Likert scales [50].

Additionally, various (mental) health variables were assessed. Body Mass Index (BMI) was calculated using weight (in kilograms) divided by height squared (in meters). Participants also rated their overall health (self-rated health) using the question, “In general, how would you rate your health?” [51]. Self-esteem was measured using the statement, “In general, I have high self-esteem” [52]. Both self-rated health and self-esteem were assessed on a five-point Likert scale.

Perceived stress was evaluated using the Perceived Stress Scale (PSS), consisting of ten items rated on a five-point Likert scale, with a range of 0–40 [53,54,55]. Irritation lies on a spectrum between normal fatigue and mental illness, and prolonged exposure to inappropriate stressors can lead to more severe conditions, such as depression [56]. The Student Irritation Scale assesses this prolonged exhaustion through six items rated on a seven-point Likert scale, effectively measuring university students’ self-reported cognitive and emotional strain [56]. Furthermore, to account for positive mental health, this survey also considered student engagement. Characterized by intrinsic motivation, active learning participation, and self-regulation skills, engaged students exhibit higher academic performance. Student engagement was assessed using the Utrecht Work Engagement Scale—Student Form (UWES-9), which comprises nine items rated on a seven-point Likert scale [57]. Anxiety and depression levels were assessed using the ultra-brief screening scale Patient Health Questionnaire (PHQ-4), which included four items rated on a four-point Likert scale [58]. Higher scores on each scale correspond to increased levels of mental distress or engagement, respectively.

### 2.3. Statistical Analysis

Descriptive statistics, including mean values, standard deviations (SD), and range, were employed to describe sample characteristics. Pearson correlation coefficients (*r*) assessed the associations between higher-order personality traits, SRF, and PA variables. The internal consistency of the scales was evaluated using Cronbach’s alpha. Multiple linear and logistic regression models were employed to examine the associations between personality traits and SRF and PA variables, as well as adherence to PA guidelines while accounting for covariates. Stepwise regression analyses were conducted to identify potential confounding variables, which were then included in the final regression models. Initial analyses examined the associations between dependent variables and identified covariates. Subsequently, the associations between individual personality traits and dependent variables were examined, independent of the other four domains and accounting for covariates. Lastly, the full model assessed the combined association of all personality traits with dependent variables, while considering the effects of identified covariates. The results of multiple linear regression models were reported as unstandardized (B) and standardized regression coefficients (*ß*), and odds ratios with 95% confidence intervals were derived from logistic regression analysis to explore the link between personality and meeting PA guidelines in both crude and adjusted models. Statistical significance was defined as *p* < 0.05. The statistical analyses in this study were conducted using SPSS version 27 (IBM Corporation, Armonk, NY, USA). Figures were built in RStudio (2023.06.0+421).

## 3. Results

A total of 4244 students provided consent and initiated their responses to the survey, resulting in an overall response rate of 10.0%. The completeness rate, a measure for attrition, was 95.5% (4054 students submitted the last questionnaire page). Participation rates varied among the 21 university departments, ranging from 7.2% to 22.1%. Among the participants, 68% identified as female students. The mean age was 23.7 ± 4.1 years, and the mean BMI was 22.8 ± 3.8 kg∙m^–2^. In terms of study workload, 25% reported dedicating 20–29 h per week to their studies, while an equal percentage reported spending 30–39 h per week. Nearly half of the students indicated having a weekly budget of up to EUR 700, approximately one-third reported being in the EUR 700–1150 range, and around 13% of students had over EUR 1150 at their disposal.

Approximately 90.5% reported themselves as non-smokers. Among the remaining 9.5%, half reported engaging in smoking either daily or occasionally. In terms of alcohol use, the mean Audit-C score was 3.1 ± 2.2, and 50.5% of the students were identified as being at an increased risk for an alcohol-related disorder (as defined by a score of four for men and three for women). When applying the cutoff for risky alcohol use (score of five for males and four for females), approximately one-third of the sample (34.2%) met this criterion. A majority (51.9%) reported consuming 1–2 servings of fruits and vegetables daily. A significant proportion (38.5%) managed to reach 3–4 servings, while only a small percentage (7.8%) met the recommended intake of at least five servings per day. The analysis of sleep quality revealed a mean score of 4.5 ± 2.2, suggesting that a significant portion of the student population (42.1%) faced challenges with their sleep quality.

The assessment of (mental) health variables revealed that 70.4% of the students perceived their health as (very) good, and a substantial majority (59.2%) of students reported having high self-esteem. The Perceived Stress Scale (PSS) yielded an average score of 19.2 ± 7.1, with 48.3% of respondents reporting increased perceived stress (score ≥ 20). The mean score for irritation was 3.9 ± 1.3 on a scale of 1–7. Student engagement had a mean score of 3.3 ± 1.1 on a scale of 0–6, and the mean score for PHQ-4 was 4.4 ± 2.9 on a scale of 0–12. An analysis of the PHQ-4 indicated that 30% of students screened positive for depressive symptoms, 33.6% screened positive for an anxiety disorder, and 28% experienced psychological distress.

A total of 4136 students completed the IPAQ-SF, yielding 4056 valid PA datasets (*n* = 80 excluded due to missing values). The BFI-2-S had valid responses from *n* = 4058 participants. Table 1 provides a detailed depiction of the survey findings, encompassing personality traits, SRF levels, MSA, and ST. In terms of aerobic PA, students accumulated an average of 2445 ± 1958 MET minutes per week, composed of vigorous PA (1145 ± 1344 MET minutes per week), moderate PA (672 ± 725 MET minutes per week), and walking activity (628 ± 823 MET minutes per week). When focusing exclusively on aerobic PA, a substantial 91.8% of students met the corresponding guidelines. However, only 30.6% of students adhered to the MSA guideline, and 27.6% adhered to the recommendation for ST. When considering all three criteria collectively, only 9.5% adhered to the current (inter-)national PA recommendations.

Table 2 presents correlation coefficients indicating the relationships between personality traits, SRF, and PA variables. Extraversion (*r =* 0.13–0.29) and Conscientiousness (*r* = 0.10–0.24) showed positive correlations with SRF, PA, and MSA. On the other hand, Open-Mindedness was not significantly correlated (*r* = −0.04–0.02), while Negative Emotionality was inversely related to SRF, PA and MSA (*r* = −0.10–−0.30). The findings for Agreeableness were mixed, yielding varied results. Associations between personality traits and ST followed a reversed pattern with negligible effects (*r* = −0.04–0.07).

Table 3 (SRF), Table 4 (PA), Table 5 (MSA), and Table 6 (ST) illustrate the associations of individual and combined personality trait domains with SRF, PA, MSA, and ST, accounting for the effects of covariates. Extraversion exhibited the strongest associations with SRF (*ß* = 0.15), PA (*ß* = 0.17), and MSA (*ß* = 0.12). The results indicate that for each unit increase in Extraversion, PA increased by 474 MET minutes per week and by 17 min of weekly MSA. Conscientiousness showed similar, albeit weaker associations (*ß* = 0.04–0.11). Negative Emotionality displayed inverse associations with SRF (*ß* = −0.06) and PA (*ß* = −0.09) individually, but the combined effects of all personality traits had minimal relationships with SFR and PA (*ß* = −0.01–−0.04). Open-Mindedness had negative and more robust associations with SFR (*ß* = −0.04–−0.08) compared to Negative Emotionality. Agreeableness yielded mixed results. Lastly, ST demonstrated negligible associations with personality trait domains (Table 6).

Table S1 provides an overview of the associations between personality trait domains and aerobic PA intensities (vPA, mPA, and walking) based on correlation coefficients. Controlling for covariates, the findings revealed noticeable intensity-dependent relationships for Extraversion, as well as subtle intensity-dependent relationships for Negative Emotionality and Conscientiousness. Stronger associations were observed at higher PA intensities. For example, each unit increase in Extraversion was associated with an additional 301 vigorous MET minutes per week (Table S2), 125 moderate MET minutes per week (Table S3), and 75 walking MET minutes per week (Table S4). Open-Mindedness showed a positive correlation with low PA intensity (walking) but a negative correlation with high PA intensity (vPA, Figure S1). After adjusting for confounding variables, only Extraversion remained significantly associated with all three PA intensities (*ß* = 0.07–0.16). Open-Mindedness exhibited significant associations with vPA (*ß* = −0.09) and walking (*ß* = 0.04). The remaining trait domains showed insignificant and trivial associations.

**Figure 1:**
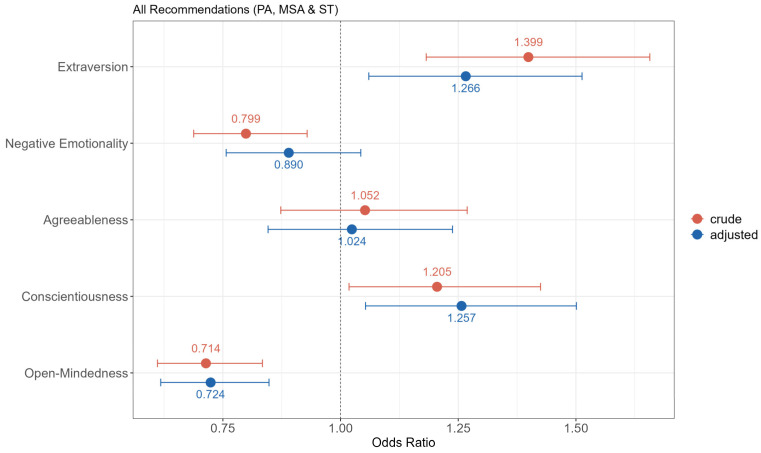
Association between personality traits and meeting all PA recommendations (PA = aerobic physical activity guideline, MSA = muscle-strengthening activity guideline, ST = sitting time recommendation). Bars represent odds ratios and corresponding 95% confidence intervals for crude and adjusted models (covariates: study workload, self-rated health, alcohol use, and fruit and vegetable consumption).

Regarding adherence to PA guidelines, Extraversion and Conscientiousness showed positive associations, indicating a higher likelihood of meeting the guidelines. Conversely, students with high Open-Mindedness demonstrated significantly lower odds of meeting the guidelines. Negative Emotionality initially showed an inverse association with guideline adherence in the crude model, but this association diminished after adjusting for covariates. Notably, Agreeableness consistently exhibited no association with adherence to the guidelines (Figure 1).

## 4. Discussion

This study examined the relationship between the ‘Big Five’ personality traits and SRF, various PA variables, and adherence to current PA guidelines among German university students. Correlation analyses largely supported the initial hypothesis, revealing generally weak but positive associations with Extraversion and Conscientiousness, inverse associations with Negative Emotionality, and no correlation with Agreeableness. Contrary to the first hypothesis, Open-Mindedness showed no association with SRF and PA. Most associations remained significant even after controlling for confounding variables, providing support for the second hypothesis. However, after accounting for covariates, the association between Negative Emotionality and MSA became non-significant, while ST appeared unrelated to personality traits in this sample. Considering activity intensities (vPA, mPA, and walking), the findings indicate that as intensity increases, the associations with Extraversion strengthen, supporting the third hypothesis. Notably, even after controlling for covariates, both Extraversion and Conscientiousness were positively associated with meeting current PA guidelines encompassing aerobic PA, MSA, and ST recommendations. Surprisingly, a robust inverse association between adherence to PA guidelines and Open-Mindedness was found but only modest and non-significant associations with Negative Emotionality in the adjusted model, contradicting the final study hypothesis.

Empirical evidence linking fitness and personality remains limited. Nevertheless, the current findings from a representative sample of university students align with a previous cross-sectional study involving 515 adolescents aged 15–17 years. In that study, Extraversion and Conscientiousness exhibited positive associations with objective measures of physical fitness (e.g., 50 m run, standing long jump, sit-up, and trunk flexion), while negative emotionality showed a negative relationship with these indicators [59]. Regarding PA, the current results further support previous meta-analyses primarily conducted with adult samples, as well as cross-sectional analyses among physical education students [37]. These studies consistently identify Extraversion, Negative Emotionality, and Conscientiousness as the most reliable correlates of PA [33,34,60,61]. However, the current sample did not confirm a significant positive relationship between PA and Open-Mindedness, as suggested by a recent meta-analysis, considering 64 studies and including 88,400 participants [34].

The observed links between higher-order personality trait domains, SRF, and PA appear robust. Extraverts typically exhibit sociability, high energy levels, and a penchant for excitement. Engaging in sports and leisure activities allows them to fulfill their social needs and seek out novel and stimulating experiences. However, it is worth noting that Introverts can also engage in PA, yet with differing motives. Howard et al. (1987) discovered that Introverts tend to gravitate towards activities such as gardening or home improvement [62]. Moreover, they may be more inclined to participate in individual sports or leisure activities, which explains the relatively modest correlations between PA and Extraversion overall. Individuals with a goal-oriented mindset and high self-control, characteristic of those scoring high on the Conscientiousness sub-trait, are likely to prioritize their health and well-being. They demonstrate greater adherence to regular PA and exercise, as they recognize the associated health benefits of an active lifestyle [60]. On the other hand, individuals with high Negative Emotionality are prone to experiencing negative effects following exercise [34], which may elucidate the stronger negative associations observed between increasing PA intensity and Negative Emotionality in the present study. Furthermore, individuals with high Negative Emotionality tend to be less emotionally stable and are more prone to distress, anxiety, and depression, increasing the likelihood of avoiding or canceling PA and exercise routines. This further accounts for the inverse relationship between PA and Negative Emotionality [60,63].

Except for Negative Emotionality, the associations between personality traits and MSA mirrored those observed for overall PA, and these significant associations remained or even strengthened after accounting for confounding variables. These findings align with expectations, as Extraverts typically engage in supervised or group-based exercise, while Conscientiousness is linked to a preference for structured activities, leading to positive associations [25,35]. Inverse associations with Open-Mindedness might be explained by a preference for outdoor activities rather than gym or home exercises [25,35]. Surprisingly, despite a significant initial correlation, the relationship between Negative Emotionality and MSA became non-significant after controlling for covariates. This finding contradicts a previous cross-sectional study among 1220 participants that found an inverse association between Negative Emotionality and muscle strength, independent of covariates [64]. A possible methodological explanation for this discrepancy is that the previous study analyzed data from a device-based strength measurement using an isokinetic dynamometer [64], whereas the current study relied on self-reported frequency and volume of MSA, which is more subjective and prone to bias influenced by social norms. Additionally, while Tolea et al. (2012) controlled for sociodemographic confounders such as age, gender, race, and BMI, they did not consider (mental) health or behavioral factors. However, there are strong correlations between Negative Emotionality and indicators of mentally ill health, such as depression, anxiety, and rumination [65], as well as unfavorable dietary choices [66], which are also associated with muscular strength and MSA [67,68,69]. Future studies should incorporate these factors to address these discrepancies and provide further clarity.

Consistent with recent meta-analyses covering >160,000 participants, Negative Emotionality and Conscientiousness demonstrated the strongest associations with daily ST [30,36]. This finding is plausible, as individuals with higher Conscientiousness tend to exhibit organizational skills and discipline, traits that likely extend to their PA behaviors. Their lower perceived barriers and higher control over engaging in PA and exercise also contribute to reduced ST [35]. Conversely, a recent meta-analysis covering 16 samples indicated that for every standard deviation increase in Negative Emotionality, the risk of physical inactivity rises by 10% [36]. Specifically, individuals with high Negative Emotionality are inclined towards sedentary pursuits, such as media consumption, and exhibit a lack of enjoyment in PA [36,70]. It is important to note that the correlation coefficients in the current study were generally small and negligible (r < 0.1). In the fully adjusted model, controlling for covariates revealed significant but modest associations only between ST and Negative Emotionality (r = 0.05). As there is a lack of comparable research, explanations remain speculative, but the results likely stem from two main factors related to methodology and study setting. Firstly, in light of a recent meta-analysis conducted by Allen et al. (2016) encompassing 28 samples, differentiating between domains of sedentary behavior could highlight the effects of personality traits on ST more effectively, considering that high Extraversion correlates with social media use but less overall ST, including watching TV or playing video games [30]. Future studies should explore this differentiation. Second, despite the large sample size, the cohort from a single setting/university must be described as rather homogeneous overall, especially since tertiary education can generally be described as a sedentary setting in which students engage in higher levels of ST during courses and at home during study periods when compared to the general population of the same age [22]. This limits the necessary variability in ST among study participants and likely contributes to the trivial effects observed between personality and ST.

Consideration of aerobic PA intensities (vPA, mPA, and walking) suggests that as intensity increases, the associations with Extraversion and—to a lesser extent—Negative Emotionality become stronger. Again, these associations are consistent with expectations, as Extraverts are typically energetic, seeking excitement, and engaged in PA, while individuals with higher levels of neuroticism, characterized by emotional instability and anxiety, tend to avoid high-intensity activities due to experiencing negative emotions. Consequently, they tend to prefer low-intensity activities or sedentary behavior [25,35]. In this study, as the intensity of PA increased, it turned the initially positive association with Open-Mindedness into a negative relationship, suggesting a dependence on activity intensity. This intensity-dependent association likely explains the absence of a link between aerobic PA and Open-Mindedness in the current sample, thus contradicting a recent meta-analysis [34]. High Open-Mindedness is associated with curiosity about different cultures or experiences, a desire for new discoveries, and a preference for diverse and novel experiences. These characteristics may align more with PA like walking for travel and sightseeing, rather than higher-intensity activities like vPA, such as running or fast swimming. Additionally, Open-Mindedness has been previously linked to outdoor activities rather than gym exercises and a preference for recreational rather than competitive PA [25,35], underscoring the importance of considering activity intensities in future studies when examining the relationship between personality and PA.

To date, no study has examined the association between personality traits and adherence to comprehensive PA guidelines encompassing aerobic PA, MSA, and avoiding prolonged ST, posing a challenge to the interpretation of the current findings. The varied and sometimes contradictory associations between personality traits and different PA variables may explain the lack of such research. Nevertheless, data on this association are highly valuable due to the significance of these guidelines for overall health and well-being. Regular PA and exercise among students can enhance their overall well-being, as well as their physical, mental, and social health, which are crucial for successful academic pursuits [71,72]. After adjusting for relevant confounding factors, the results revealed that individuals with high levels of Extraversion and Conscientiousness were more likely to meet the PA guidelines, supporting a genuine association considering the inclusion of various health-related PA variables. Equally conforming to expectations, Agreeableness proved unrelated to the adherence of PA guidelines. Interestingly, students with higher levels of Negative Emotionality showed a trend of lower adherence to the guidelines, but this association did not reach statistical significance in the adjusted model, emphasizing the importance of accounting for relevant behavioral and (mental) health confounders beyond age and sex. Most surprisingly, students with high Open-Mindedness had lower odds of meeting the PA guidelines. While direct causality cannot be assumed, several factors may contribute to this finding. For instance, individuals high in Open-Mindedness tend to be intellectually curious, imaginative, and creative, gravitating toward mentally stimulating activities that are typically sedentary and do not involve physical exercise. Additionally, with numerous commitments such as lectures, studying, social activities, and part-time jobs, individuals high in Open-Mindedness may have limited time available for regular PA and exercise.

Moving forward, future research in this field should expand upon the existing literature by delving into the lower-order facets of the ‘Big Five’ personality traits. Previous studies have predominantly focused on higher-order traits [33,34,36], potentially overlooking the nuanced associations that may exist at the facet level. It is plausible that different facets within each trait exhibit varying degrees of relationship and even distinct directions of association with PA. Consequently, the incorporation of facet-level analyses becomes crucial for a comprehensive understanding of the intricate interplay between personality and PA [60,73]. By investigating the predictive power of specific facets, researchers can ascertain whether certain facets within the ‘Big Five’ personality traits hold greater influence in determining PA levels among university students. This avenue will help uncover nuanced associations and advance our understanding of the complex relationship between personality and PA, paving the way for more targeted interventions and strategies to promote PA among this population.

### Strengths and Limitations

The present results need to be interpreted in light of the strengths and limitations of the study. First, the assessment of PA is inherently complex due to its multicomponent nature, including frequency, intensity, duration, and mode. While this study at least accounted for intensity and mode, it relied on self-reports, which are susceptible to recall bias and social desirability. In future research, the use of device-based PA monitors in conjunction with self-reports would provide more accurate and comprehensive measures of PA and allow for better discrimination between different domains of sedentary behaviors. Furthermore, although cross-sectional analyses provided valuable insights, prospective study designs are needed to establish causal inference. Finally, this is a monocentric study, which limits its generalizability to other types of higher education institutions or regions. On the other hand, this study has several notable strengths. First, it has a large and representative sample, including students from each faculty of the university. The sample consisted of students only and thus a comparatively homogeneous young group, which is crucial given the observed changes in PA and personality with age. Another strength is the thorough consideration of a wide range of potential confounding factors, which contributes to a more comprehensive understanding of the relationships under investigation. Lastly, this study analyzed multiple PA variables making up the current PA guidelines, including combined aerobic PA, MSA, and ST, which strengthens the validity and robustness of the study findings.

## 5. Conclusions

Elucidating the factors that influence students’ engagement in health-promoting behaviors, such as regular physical activity and exercise, is of paramount importance for effective university health management strategies. Although the associations between personality and PA may appear modest based on the current findings, the adoption of a facet-level approach in future research offers promising avenues for improving our understanding of the intricate relationship between personality and PA. Identifying the most effective interventions tailored to individuals or groups with specific characteristics (e.g., personality traits) paves the way for personalized interventions. Such strategies hold promise for promoting beneficial health behaviors such as PA engagement among students, ultimately leading to improved overall health and well-being within the university community.

## Figures and Tables

**Table 1:** Descriptive statistics for personality trait domains, self-rated fitness, muscle-strengthening activity, and sitting time.

Variable	Mean ± SD	Range
Extraversion	3.20 ± 0.70	1–5
Negative Emotionality	2.85 ± 0.79	1–5
Agreeableness	3.83 ± 0.60	1–5
Conscientiousness	3.64 ± 0.68	1–5
Open-Mindedness	3.56 ± 0.70	1.17–5
Self-rated fitness	3.29 ± 0.95	1–5
Muscle-strengthening activity (sessions per week)	1.25 ± 1.50	0–7
Sitting time (hours per day)	9.35 ± 4.95	0–24

**Table 2:** Pearson correlation coefficients for the association between personality traits and fitness/ physical activity variables.

	Extraversion	Negative Emotionality	Agreeableness	Conscientiousness	Open-Mindedness	SRF	PA	MSA	ST
Extraversion	1								
Negative Emotionality	−0.33 ***	1							
Agreeableness	0.14 ***	−0.24 ***	1						
Conscientiousness	0.24 ***	−0.22 ***	0.19 ***	1					
Open-Mindedness	0.22 ***	−0.15 ***	0.08 ***	0.06 ***	1				
SRF	0.29 ***	−0.30 ***	0.11 ***	0.24 ***	0.02	1			
PA	0.20 ***	−0.13 ***	0.04 *	0.10 ***	0.02	0.38 ***	1		
MSA	0.13 ***	−0.10 ***	−0.03	0.10 ***	−0.04 *	0.35 ***	0.46 ***	1	
ST	−0.05 ***	0.07 ***	−0.04	−0.06 ***	0	−0.12 ***	−0.07 ***	−0.06 **	1

*** indicates *p* < 0.001, ** indicates *p* < 0.01, * indicates *p* < 0.05; SRF = self-rated fitness, PA = physical activity, MSA = muscle-strengthening activities, ST = sitting time.

**Table 3:** Association between personality traits and self-rated fitness (5-point Likert scale) after controlling for covariates.

Variable	Model 1		Model 2		Model 3		Model 4		Model 5		Model 6		Model 7	
	B	*ß*	B	*ß*	B	*ß*	B	*ß*	B	*ß*	B	*ß*	B	*ß*
Intercept	1.74		1.38		2.15		1.54		1.07		1.80		1.12	
Self-rated Health	0.34	0.31 ***	0.33	0.30 ***	0.33	0.30 ***	0.34	0.31 ***	0.33	0.30 ***	0.34	0.31 ***	0.31	0.28 ***
Self-Esteem	0.17	0.17 ***	0.12	0.12 ***	0.14	0.14 ***	0.17	0.17 ***	0.15	0.15 ***	0.17	0.18 ***	0.10	0.10 ***
Body Mass Index	−0.03	−0.14 ***	−0.04	−0.14 ***	−0.03	−0.14 ***	−0.03	−0.13 ***	−0.03	−0.12 ***	−0.03	−0.13 ***	−0.03	−0.13 ***
Fruit and Vegetable Intake	0.18	0.13 ***	0.16	0.11 ***	0.18	0.12 ***	0.18	0.12 ***	0.17	0.12 ***	0.18	0.13 ***	0.16	0.11 ***
Sex (f = 0, m = 1)	0.18	0.09 ***	0.23	0.11 ***	0.16	0.08 ***	0.20	0.10 ***	0.22	0.11 ***	0.19	0.09 ***	0.26	0.13 ***
Alcohol Use	0.02	0.06 ***	0.01	0.02	0.02	0.05 ***	0.02	0.06 ***	0.03	0.07 ***	0.02	0.05 ***	0.02	0.03 *
Age	−0.01	−0.03 *	−0.01	−0.03	−0.01	−0.03 *	−0.01	−0.03	−0.01	−0.03 *	−0.01	−0.03	−0.01	−0.03
Extraversion			0.22	0.16 ***									0.20	0.15 ***
Negative Emotionality					−0.08	−0.06 ***							−0.02	−0.02
Agreeableness							0.06	0.03 *					0.02	0.01
Conscientiousness									0.19	0.14 ***			0.15	0.11 ***
Open-Mindedness											−0.03	−0.02	−0.08	−0.06 ***

Model 1 examines the effect of the covariate set on self-rated fitness, Models 2 to 6 add a single personality trait to Model 1 (Model 2 = Extraversion, Model 3 = Negative Emotionality, Model 4 = Agreeableness, Model 5 = Conscientiousness, and Model 6 = Open-Mindedness); Model 7 adds all personality traits to Model 1. *** indicates *p* < 0.001, * indicates *p* < 0.05.

**Table 4:** Association between personality traits and self-reported physical activity (MET minutes per week) after controlling for covariates.

Variable	Model 1		Model 2		Model 3		Model 4		Model 5		Model 6		Model 7	
	B	*ß*	B	*ß*	B	*ß*	B	*ß*	B	*ß*	B	*ß*	B	*ß*
Intercept	267.8		−811.0		916.4		120.1		−300.0		347.5		−404.8	
Fruit and Vegetable Intake	396.0	0.13 ***	354.9	0.12 ***	388.2	0.13 ***	394.9	0.13 ***	380.9	0.13 ***	399.5	0.13 ***	359.4	0.12 ***
Self-rated Health	229.3	0.10 ***	204.7	0.09 ***	219.6	0.10 ***	228.7	0.10 ***	218.1	0.10 ***	228.3	0.10 ***	188.8	0.08 ***
Sex (f = 0, m = 1)	234.8	0.06 ***	302.7	0.07 ***	182.6	0.04 **	246.4	0.06 ***	279.3	0.07 ***	237.1	0.06 ***	311.8	0.07 ***
Sleep Quality	41.8	0.05 ***	47.5	0.05 ***	49.8	0.06 **	41.5	0.05 **	47.4	0.05 **	42.1	0.05 **	56.2	0.06 ***
Irritation	−11.1	−0.05 ***	−9.2	−0.04 *	−2.16	−0.01	−10.4	−0.04 *	−12.1	−0.05 **	−11.3	−0.05 **	−6.6	−0.03
Self-Esteem	88.1	0.04 *	−12.5	−0.01	29.5	0.01	86.9	0.04 *	61.4	0.03	90.1	0.04	−45.0	−0.02
Extraversion			480.4	0.17 ***									473.6	0.17 ***
Negative Emotionality					−216.4	−0.09 ***							−102.0	−0.04
Agreeableness							36.0	0.01					−14.3	0
Conscientiousness									199.4	0.07 ***			125.7	0.04 **
Open-Mindedness											−25.5	−0.01	−122.9	−0.04 **

Model 1 examines the effect of the covariate set on self-reported physical activity, and Models 2 to 6 add a single personality trait to Model 1 (Model 2 = Extraversion, Model 3 = Negative Emotionality, Model 4 = Agreeableness, Model 5 = Conscientiousness, and Model 6 = Open-Mindedness); Model 7 adds all personality traits to Model 1. *** indicates *p* < 0.001, ** indicates *p* < 0.01, * indicates *p* < 0.05.

**Table 5:** Association between personality traits and self-reported muscle-strengthening activity (days per week by minutes per session) after controlling for covariates.

Variable	Model 1		Model 2		Model 3		Model 4		Model 5		Model 6		Model 7	
	B	*ß*	B	*ß*	B	*ß*	B	*ß*	B	*ß*	B	*ß*	B	*ß*
Intercept	−9.2		−39.4		9.8		12.1		−45.0		12.1		−10.9	
Sex (f = 0, m = 1)	31.3	0.14 ***	34.1	0.15 ***	30.1	0.14 ***	29.5	0.13 ***	34.6	0.16 ***	31.8	0.14 ***	35.3	0.16 ***
Fruit and Vegetable Intake	19.1	0.12 ***	17.9	0.12 ***	19.0	0.12 ***	19.3	0.13 ***	18.3	0.12 ***	20.2	0.13 ***	18.5	0.12 ***
Self-rated Health	11.4	0.10 ***	10.6	0.09 ***	11.0	−0.09 ***	11.6	0.10 ***	10.8	0.09 ***	11.0	0.09 ***	9.4	0.08 ***
Age	−1.6	−0.06 ***	−1.6	−0.06 ***	−1.6	−0.07 ***	−1.6	−0.07 **	−1.6	−0.07 ***	−1.5	−0.06 ***	1.4	−0.06 **
Student Engagement	−4.6	−0.05 **	−6.2	−0.07 ***	−4.9	−0.05 **	−4.3	−0.05 **	−6.1	−0.07 ***	−3.0	−0.03	5.5	−0.06 *
Self-Esteem	5.6	0.05 *	2.1	0.02	4.3	0.04	5.9	0.06 **	4.0	0.04	5.9	0.06 **	1.1	0.01
Extraversion			16.8	0.12 ***									17.3	0.12 ***
Negative Emotionality					−3.4	−0.03							−1.5	−0.01
Agreeableness							−6.1	−0.04 *					−8.0	−0.05 *
Conscientiousness									13.9	0.09 ***			12.2	0.08 ***
Open-Mindedness											−8.7	−0.06 ***	−11.0	−0.08 ***

Model 1 examines the effect of the covariate set on self-reported muscle-strengthening activity, Models 2 to 6 add a single personality trait to Model 1 (Model 2 = Extraversion, Model 3 = Negative Emotionality, Model 4 = Agreeableness, Model 5 = Conscientiousness, and Model 6 = Open-Mindedness); Model 7 adds all personality traits to Model 1. *** indicates *p* < 0.001, ** indicates *p* < 0.01, * indicates *p* < 0.05.

**Table 6:** Association between personality traits and self-reported sitting time (hours) after controlling for covariates.

Variable	Model 1		Model 2		Model 3		Model 4		Model 5		Model 6		Model 7	
	B	*ß*	B	*ß*	B	*ß*	B	*ß*	B	*ß*	B	*ß*	B	*ß*
Intercept	10.66		10.68		11.45		11.25		11.22		10.23		12.42	
Anxiety and Depression	0.13	0.08 ***	0.13	0.08 ***	0.17	0.10 ***	0.13	0.08 ***	0.13	0.07 ***	0.13	0.08 ***	0.16	0.10 ***
Self-Esteem	−0.33	−0.06 ***	−0.33	−0.06 ***	−0.39	−0.08 ***	−0.32	−0.06 ***	−0.30	−0.06 **	−0.34	−0.07 ***	−0.37	−0.07 ***
Fruit and Vegetable Intake	−0.28	−0.04 *	−0.28	−0.04 *	−0.29	−0.04 *	−0.27	−0.04 *	−0.26	−0.04 *	−0.30	−0.04 *	−0.27	−0.04 *
Extraversion			−0.01	0									−0.02	0
Negative Emotionality					−0.26	−0.04							−0.29	−0.05 *
Agreeableness							−0.17	−0.02					−0.20	−0.02
Conscientiousness									−0.19	−0.03			−0.18	−0.02
Open-Mindedness											0.14	0.02	0.13	0.02

Model 1 examines the effect of the covariate set on self-reported sitting time, Models 2 to 6 add a single personality trait to Model 1 (Model 2 = Extraversion, Model 3 = Negative Emotionality, Model 4 = Agreeableness, Model 5 = Conscientiousness, and Model 6 = Open-Mindedness); Model 7 adds all personality traits to Model 1. *** indicates *p* < 0.001, ** indicates *p* < 0.01, * indicates *p* < 0.05.

## Data Availability

The data presented in this study are available on reasonable request.

## Supplementary Materials

The following supporting information can be downloaded at: https://www.mdpi.com/article/10.3390/ejihpe13080104/s1, Figure S1: Association between personality traits and physical activity intensity variables (vPA = vigorous physical activity, mPA = moderate physical activity); bars represent unstandardized regression coefficient and corresponding 95% confidence intervals for adjusted models (covariates: compare Tables S2–S4) in MET per week; Table S1: Pearson correlation coefficients for the association between personality traits and physical activity variables derived from IPAQ-SF; Table S2: Association between personality traits and self-reported vigorous physical activity after controlling for covariates; Table S3: Association between personality traits and self-reported moderate physical activity after controlling for covariates; Table S4: Association between personality traits and self-reported walking after controlling for covariates.
