# Supplementary material for: Personality Traits and Physical Activity: Insights from German University Students
Source: Eur J Investig Health Psychol Educ. 2023 Aug 3;13(8):1423–40. doi: 10.3390/ejihpe13080104 (PMC10453150; doi:10.3390/ejihpe13080104)
Supplement: Supplementary file 1 [file ejihpe-13-00104-s001.zip › ejihpe-2519624_Supplements.pdf]

European Journal of
*Investigation in Health, Psychology and Education*

*Article*

# Personality Traits and Physical Activity: Insights from German University Students

**Carsten Müller**

**Table S1.** Pearson correlation coefficients for the association between personality traits and physical activity variables derived from IPAQ-SF

| | Extraversion | Negative Emotionality | Agreeableness | Conscientiousness | Open-Mindedness | vPA | mPA | walk |
|---|---|---|---|---|---|---|---|---|
| Extraversion | 1 | | | | | | | |
| Negative Emotionality | -0.33 *** | 1 | | | | | | |
| Agreeableness | 0.14 *** | -0.24 *** | 1 | | | | | |
| Conscientiousness | 0.24 *** | -0.22 *** | 0.19 *** | 1 | | | | |
| Open-Mindedness | 0.22 *** | -0.15 *** | 0.08 *** | 0.06 *** | 1 | | | |
| vPA | 0.18 *** | -0.14 *** | 0.01 | 0.08 *** | -0.03 | 1 | | |
| mPA | 0.16 *** | -0.09 *** | 0.05 *** | 0.08 *** | 0.04 | 0.28 *** | 1 | |
| walk | 0.04 * | -0.01 | 0.02 | 0.03 | 0.07 *** | 0.02 | 0.20 *** | 1 |

*** indicates $p < 0.001$, ** indicates $p < 0.01$, * indicates $p < 0.05$; vPA = vigorous physical activity, mPA = moderate physical activity

**Table S2.** Association between personality traits and self-reported vigorous physical activity after controlling for covariates

| Variable | Model 1 | | Model 2 | | Model 3 | | Model 4 | | Model 5 | | Model 6 | | Model 7 | |
|---|---|---|---|---|---|---|---|---|---|---|---|---|---|---|
| | **B** | ***ß*** | **B** | ***ß*** | **B** | ***ß*** | **B** | ***ß*** | **B** | ***ß*** | **B** | ***ß*** | **B** | ***ß*** |
| Intercept | -124.5 | | -768.6 | | 360.9 | | -46.5 | | -351.3 | | 149.7 | | -16.6 | |
| Self-rated Health | 267.5 | 0.17 *** | 213.8 | 0.14 *** | 229.7 | 0.15 *** | 269.1 | 0.17 *** | 255.9 | 0.16 *** | 269.0 | 0.17 *** | 195.1 | 0.12 *** |
| Fruit and Vegetable Intake | 229.1 | 0.11 *** | 203.2 | 0.10 *** | 222.6 | 0.11 *** | 230.2 | 0.11 *** | 223.8 | 0.11 *** | 243.4 | 0.12 *** | 221.9 | 0.11 *** |
| Sex (f=0, m=1) | 232.0 | 0.08 *** | 269.6 | 0.09 *** | 195.6 | 0.07 *** | 225.7 | 0.08 *** | 248.9 | 0.09 *** | 240.5 | 0.08 *** | 261.6 | 0.09 *** |
| Age | -12.5 | -0.04 * | -9.8 | -0.03 | -13.1 | -0.04 * | -12.7 | -0.04 * | -12.5 | -0.04 * | -11.7 | -0.04 * | -8.0 | -0.02 |
| Income | -37.3 | -0.04 * | -57.3 | -0.05 * | -40.1 | -0.04 * | -38.0 | -0.04 * | -38.9 | -0.04 * | -34.7 | -0.03 | -57.3 | -0.05 ** |
| Extraversion | | | 279.1 | 0.15 *** | | | | | | | | | 301.1 | 0.16 *** |
| Negative Emotionality | | | | | -102.6 | -0.06 *** | | | | | | | -49.5 | -0.03 |
| Agreeableness | | | | | | | -20.8 | -0.01 | | | | | -57.3 | -0.03 |
| Conscientiousness | | | | | | | | | 77.7 | 0.04 * | | | 28.1 | 0.01 |
| Open-Mindedness | | | | | | | | | | | -97.6 | -0.05 ** | -163.6 | -0.09 *** |

Model 1 examines the effect of the covariate set on self-reported vigorous physical activity, Models 2 to 6 add a single personality trait to Model 1 (Model 2 = Extraversion, Model 3 = Negative Emotionality, Model 4 = Agreeableness, Model 5 = Conscientiousness, Model 6 = Open-Mindedness); Model 7 adds all personality traits to Model 1. *** indicates $p < 0.001$, ** indicates $p < 0.01$, * indicates $p < 0.05$

**Table S3.** Association between personality traits and self-reported moderate physical activity after controlling for covariates

| Variable | Model 1 | | Model 2 | | Model 3 | | Model 4 | | Model 5 | | Model 6 | | Model 7 | |
|---|---|---|---|---|---|---|---|---|---|---|---|---|---|---|
| | **B** | ***ß*** | **B** | ***ß*** | **B** | ***ß*** | **B** | ***ß*** | **B** | ***ß*** | **B** | ***ß*** | **B** | ***ß*** |
| Intercept | 87.8 | | -144.8 | | 291.5 | | -38.5 | | -81.8 | | 8.2 | | -184.2 | |
| Fruit and Vegetable Intake | 83.2 | 0.08 *** | 67.3 | 0.06 *** | 82.9 | 0.08 *** | 80.5 | 0.07 *** | 77.6 | 0.07 *** | 80.1 | 0.07 *** | 64.4 | 0.06 *** |
| Self-Esteem | 49.6 | 0.07 *** | 19.3 | 0.03 | 32.9 | 0.04 * | 46.9 | 0.06 *** | 41.6 | 0.06 ** | 47.4 | 0.06 *** | 9.0 | 0.01 |
| Alcohol Use | 15.7 | 0.05 ** | 7.0 | 0.02 | 15.1 | 0.05 ** | 16.3 | 0.05 ** | 18.7 | 0.06 *** | 16.1 | 0.05 ** | 9.3 | 0.03 |
| Subjective Health | 39.3 | 0.05 * | 32.9 | 0.04 * | 33.6 | 0.04 * | 37.6 | 0.05 * | 35.0 | 0.04 * | 40.2 | 0.05 * | 27.8 | 0.03 |
| Extraversion | | | 134.8 | 0.13 *** | | | | | | | | | 124.5 | 0.12 *** |
| Negative Emotionality | | | | | -42.1 | -0.05 * | | | | | | | -17.9 | -0.02 |
| Agreeableness | | | | | | | 38.5 | 0.03 * | | | | | 17.1 | 0.01 |
| Conscientiousness | | | | | | | | | 60.4 | 0.06 *** | | | 32.5 | 0.03 |
| Open-Mindedness | | | | | | | | | | | 25.6 | 0.02 | -1.2 | 0 |

Model 1 examines the effect of the covariate set on moderate physical activity, Models 2 to 6 add a single personality trait to Model 1 (Model 2 = Extraversion, Model 3 = Negative Emotionality, Model 4 = Agreeableness, Model 5 = Conscientiousness, Model 6 = Open-Mindedness); Model 7 adds all personality traits to Model 1. *** indicates $p < 0.001$, ** indicates $p < 0.01$, * indicates $p < 0.05$

**Table S4.** Association between personality traits and self-reported walking after controlling for covariates

| Variable | Model 1 | | Model 2 | | Model 3 | | Model 4 | | Model 5 | | Model 6 | | Model 7 | |
|---|---|---|---|---|---|---|---|---|---|---|---|---|---|---|
| | **B** | ***ß*** | **B** | ***ß*** | **B** | ***ß*** | **B** | ***ß*** | **B** | ***ß*** | **B** | ***ß*** | **B** | ***ß*** |
| Intercept | 232.9 | | 1.2 | | 287.2 | | 173.1 | | 81.8 | | 46.8 | | -187.7 | |
| Age | 21.7 | 0.11 *** | 22.7 | 0.12 *** | 21.7 | 0.11 *** | 21.9 | 0.11 *** | 22.1 | 0.11 *** | 21.3 | 0.11 *** | 22.3 | 0.11 *** |
| Fruit and Vegetable Intake | 71.7 | 0.06 *** | 60.7 | 0.05 ** | 71.1 | 0.06 *** | 70.5 | 0.06 *** | 67.6 | 0.06 *** | 63.9 | 0.05 ** | 54.8 | 0.04 ** |
| Alcohol Use | -21.3 | -0.06 *** | -26.7 | -0.07 *** | -21.6 | -0.06 *** | -21.1 | -0.06 *** | -19.0 | -0.05 ** | -20.3 | -0.05 *** | -23.6 | -0.06 *** |
| Sleep Quality | 20.9 | 0.06 ** | 23.1 | 0.06 *** | 22.4 | 0.06 ** | 21.0 | 0.06 ** | 22.5 | 0.06 *** | 20.8 | 0.06 ** | 23.2 | 0.06 *** |
| Irritation | -33.3 | -0.06 ** | -29.3 | -0.05 ** | -26.3 | -0.04 * | -32.1 | -0.05 ** | -34.2 | -0.06 ** | -30.9 | -0.05 ** | -30.8 | -0.05 * |
| Income | -27.0 | -0.04 * | -31.9 | -0.05 ** | -27.1 | -0.04 * | -26.4 | -0.04 * | -27.5 | -0.04 * | -28.8 | -0.05 * | -33.0 | -0.05 ** |
| Self-rated Health | -35.4 | -0.04 * | -47.4 | -0.05 ** | -39.4 | -0.04 * | -35.7 | -0.04 * | -41.3 | -0.04 * | -35.2 | -0.04 * | -47.9 | -0.05 ** |
| Extraversion | | | 90.1 | 0.08 *** | | | | | | | | | 75.2 | 0.07 ** |
| Negative Emotionality | | | | | -24.5 | -0.02 | | | | | | | 6.5 | 0.01 |
| Agreeableness | | | | | | | 13.8 | 0.01 | | | | | -4.2 | 0 |
| Conscientiousness | | | | | | | | | 45.5 | 0.04 * | | | 28.9 | 0.02 |
| Open-Mindedness | | | | | | | | | | | 58.5 | 0.05 ** | 43.0 | 0.04 * |

Model 1 examines the effect of the covariate set on self-reported walking, Models 2 to 6 add a single personality trait to Model 1 (Model 2 = Extraversion, Model 3 = Negative Emotionality, Model 4 = Agreeableness, Model 5 = Conscientiousness, Model 6 = Open-Mindedness); Model 7 adds all personality traits to Model 1. *** indicates $p < 0.001$, ** indicates $p < 0.01$, * indicates $p < 0.05$

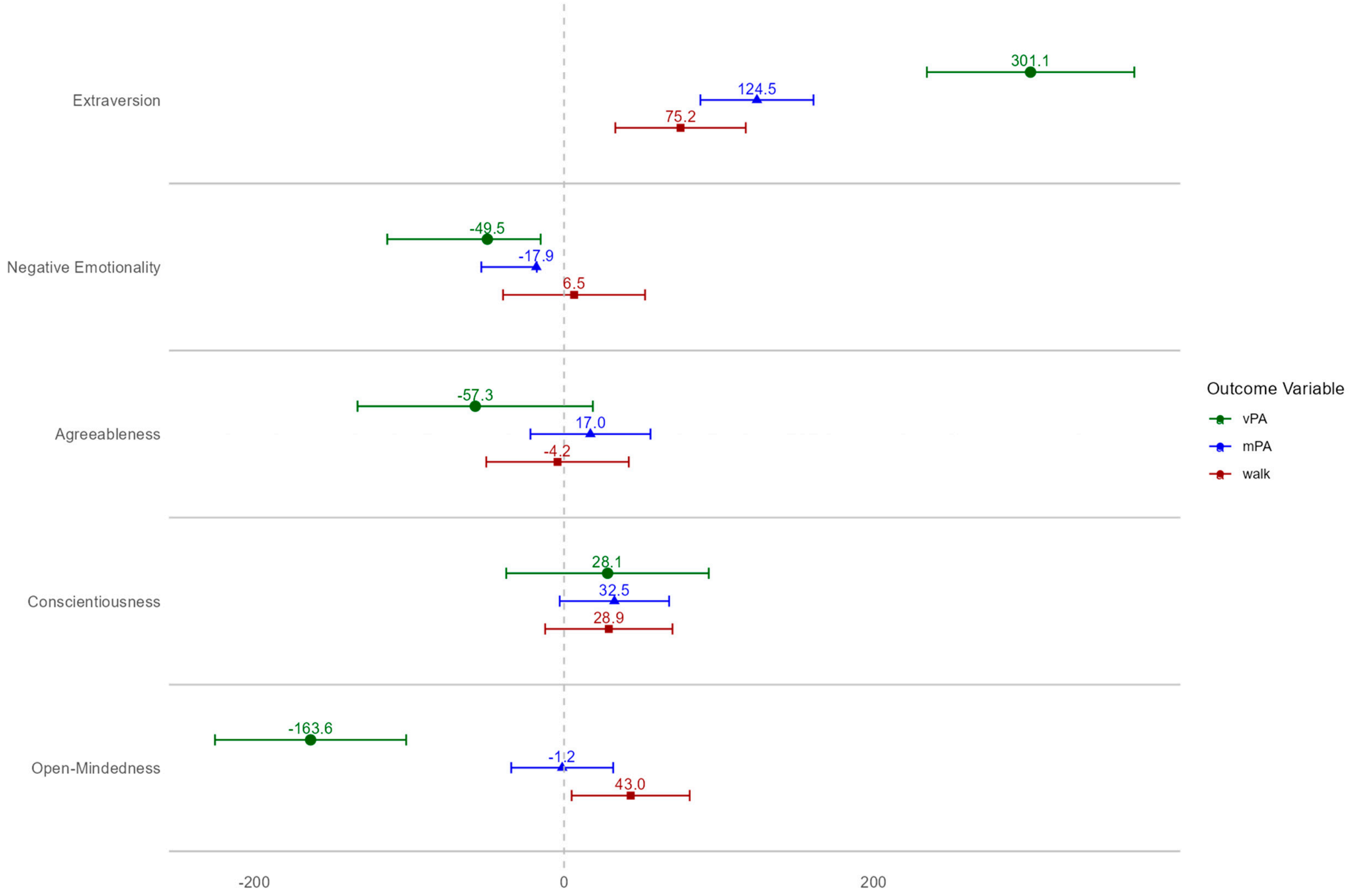


**Figure S1.** Association between personality traits and physical activity intensity variables (vPA = vigorous physical activity, mPA = moderate physical activity); Bars represent unstandardized regression coefficient and corresponding 95% confidence intervals for adjusted models (covariates: compare tables S2 – S4) in MET per week
