# Supplementary material for: Personality Traits and Physical Activity: Insights from German University Students
Source: Eur J Investig Health Psychol Educ. 2023 Aug 3;13(8):1423–40. doi: 10.3390/ejihpe13080104 (PMC10453150; doi:10.3390/ejihpe13080104)
Supplement: Supplementary file 1 [file ejihpe-13-00104-s001.zip › ejihpe-2519624_Supplements_CHERRIES.pdf]

CHERRIES Statement - Checklist for Reporting Results of Internet E-Surveys

Category	*Checklist Item*	*Explanation*	*Page Number*
Design			
	Describe survey design	Describe target population, sample frame. Is the sample a convenience sample? (In "open" surveys this is most likely.)<br>"This is an online cross-sectional study conducted in a convenience sample at the University of Münster, Germany."	3, ll. 140-1
IRB (Institutional Review Board) approval and informed consent process			
	IRB approval	Mention whether the study has been approved by an IRB.<br>"The University Institutional Review Committee (2019-07-TU) granted ethical approval."	3, ll. 148-9
	Informed consent	Describe the informed consent process. Where were the participants told the length of time of the survey, which data were stored and where and for how long, who the investigator was, and the purpose of the study?<br>"Prior to continuing with the study, all students were required to provide mandatory in-formed consent."	4, ll. 157-8
	Data protection	If any personal information was collected or stored, describe what mechanisms were used to protect unauthorized access.<br>Each student received a unique transaction number along with the invitation email, which provided details on the estimated survey duration (20 to 30 minutes), voluntary participation, anonymity, data protection, and incentives (e.g., a chance to win VIP tickets for sporting events).	4, ll. 153-6
Development and pre-testing			
	Development and testing	State how the survey was developed, including whether the usability and technical functionality of the electronic questionnaire had been tested before fielding the questionnaire.<br>"The survey was administered using evasys version 8.0 with adaptive questioning (evasys GmbH, Lüneburg, Germany), without randomization of items. Participants had the op-tion to modify their answers on previous pages by using the back button."	4, ll. 158-162
	Open survey versus closed survey	An "open survey" is a survey open for each visitor of a site, while a closed survey is only open to a sample which the investigator knows (password-protected survey).<br>Closed survey: "Each student received a unique transaction number along with the invitation email…"	4, ll. 153-4

	Contact mode	Indicate whether or not the initial contact with the potential participants was made on the Internet. (Investigators may also send out questionnaires by mail and allow for Web-based data entry.)<br>"42,630 students received email invitations to participate."	3,4, ll. 151-1
	Advertising the survey	How/where was the survey announced or advertised? Some examples are offline media (newspapers), or online (mailing lists – If yes, which ones?) or banner ads (Where were these banner ads posted and what did they look like?). It is important to know the wording of the announcement as it will heavily influence who chooses to participate. Ideally the survey announcement should be published as an appendix.<br>Apart from the invitation e-mail, no other advertising was placed: "42,630 students received email invitations to participate."	3,4, ll. 151-1
Survey administration			
	Web/E-mail	State the type of e-survey (eg, one posted on a Web site, or one sent out through e-mail). If it is an e-mail survey, were the responses entered manually into a database, or was there an automatic method for capturing responses?<br>⇨ Sent out through e-mail: "42,630 students received email invitations to participate."<br>⇨ automatic method for capturing responses: "The survey was administered using evasys version 8.0 with adaptive questioning (evasys GmbH, Lüneburg, Germany), without randomization of items. […] There was no completeness check, but incomplete surveys were also included."	3,4, ll. 151-1<br>4, ll. 158-62
	Context	Describe the Web site (for mailing list/newsgroup) in which the survey was posted. What is the Web site about, who is visiting it, what are visitors normally looking for? Discuss to what degree the content of the Web site could pre-select the sample or influence the results. For example, a survey about vaccination on a anti-immunization Web site will have different results from a Web survey conducted on a government Web site<br>No pre-selection of the sample: "The study included all regular university students, excluding cross-registered students, auditing students, and senior citizens. 42,630 students received email invitations to participate."	3, ll. 149-51
	Mandatory/voluntary	Was it a mandatory survey to be filled in by every visitor who wanted to enter the Web site, or was it a voluntary survey?<br>Voluntary survey: "Each student received […] which provided details on the estimated survey duration (20 to 30 minutes), voluntary participation, anonymity, data protection, and incentives…"	4, ll. 153-6

	Incentives	Were any incentives offered (eg, monetary, prizes, or non-monetary incentives such as an offer to provide the survey results)?<br>"Each student received a unique transaction number along with the invitation email, which provided details on […] incentives (e.g., a chance to win VIP tickets for sporting events).	4, ll. 153-6
	Time/Date	In what timeframe were the data collected?<br>Two weeks: "Non-respondents received two reminders within a two-week period."	4, ll. 156-7
	Randomization of items or questionnaires	To prevent biases items can be randomized or alternated.<br>"The survey was administered using evasys version 8.0 with adaptive questioning (evasys GmbH, Lüneburg, Germany), without randomization of items."	4, ll. 158-60
	Adaptive questioning	Use adaptive questioning (certain items, or only conditionally displayed based on responses to other items) to reduce number and complexity of the questions.<br>"The survey was administered using evasys version 8.0 with adaptive questioning (evasys GmbH, Lüneburg, Germany), without randomization of items."	4, ll. 158-60
	Number of Items	What was the number of questionnaire items per page? The number of items is an important factor for the completion rate.<br>The number of items varied across the 15 pages. With a total of 172 items, the mean number of items per page was 11-12.<br>"The questionnaire consisted of 172 items spread across 15 pages…"	4, ll. 151-2
	Number of screens (pages)	Over how many pages was the questionnaire distributed? The number of items is an important factor for the completion rate.<br>"The questionnaire consisted of 172 items spread across 15 pages…"	4, ll. 151-2
	Completeness check	It is technically possible to do consistency or completeness checks before the questionnaire is submitted. Was this done, and if "yes", how (usually JAVAScript)? An alternative is to check for completeness after the questionnaire has been submitted (and highlight mandatory items). If this has been done, it should be reported. All items should provide a non-response option such as "not applicable" or "rather not say", and selection of one response option should be enforced.<br>"There was no completeness check, but incomplete surveys were also included."	4, ll. 161-2
	Review step	State whether respondents were able to review and change their answers (eg, through a Back button or a Review step which displays a summary of the responses and asks the respondents if they are correct).<br>"Participants had the option to modify their answers on previous pages by using the back button."	4, ll. 160-1

Response rates			
	Unique site visitor	If you provide view rates or participation rates, you need to define how you determined a unique visitor. There are different techniques available, based on IP addresses or cookies or both.<br>"Each student received a unique transaction number along with the invitation email,…"	4, ll. 153-4
	View rate (Ratio of unique survey visitors/unique site visitors)	Requires counting unique visitors to the first page of the survey, divided by the number of unique site visitors (not page views!). It is not unusual to have view rates of less than 0.1 % if the survey is voluntary.<br>Not applicable	
	Participation rate (Ratio of unique visitors who agreed to participate/unique first survey page visitors)	Count the unique number of people who filled in the first survey page (or agreed to participate, for example by checking a checkbox), divided by visitors who visit the first page of the survey (or the informed consents page, if present). This can also be called "recruitment" rate.<br>Not applicable	
	Completion rate (Ratio of users who finished the survey/users who agreed to participate)	The number of people submitting the last questionnaire page, divided by the number of people who agreed to participate (or submitted the first survey page). This is only relevant if there is a separate "informed consent" page or if the survey goes over several pages. This is a measure for attrition. Note that "completion" can involve leaving questionnaire items blank. This is not a measure for how completely questionnaires were filled in. (If you need a measure for this, use the word "completeness rate".)<br>"Completeness rate, a measure for attrition, was 95.5% (4,054 students submitted the last questionnaire page)."	5, ll. 277-8
Preventing multiple entries from the same individual			
	Cookies used	Indicate whether cookies were used to assign a unique user identifier to each client computer. If so, mention the page on which the cookie was set and read, and how long the cookie was valid. Were duplicate entries avoided by preventing users access to the survey twice; or were duplicate database entries having the same user ID eliminated before analysis? In the latter case, which entries were kept for analysis (eg, the first entry or the most recent)?<br>Not applicable (unique transaction numbers used)	
	IP check	Indicate whether the IP address of the client computer was used to identify potential duplicate entries from the same user. If so, mention the period of time for which no two entries from the same IP address were allowed (eg, 24 hours). Were duplicate entries avoided by preventing users with the same IP address access to the survey twice; or were duplicate database entries having the same IP address within a given period of time eliminated before analysis? If the latter, which entries were kept for analysis (eg, the first entry or the most recent)?<br>Not applicable (unique transaction numbers used)	

	Log file analysis	Indicate whether other techniques to analyze the log file for identification of multiple entries were used. If so, please describe.<br>Not applicable (unique transaction numbers used)	
	Registration	In "closed" (non-open) surveys, users need to login first and it is easier to prevent duplicate entries from the same user. Describe how this was done. For example, was the survey never displayed a second time once the user had filled it in, or was the username stored together with the survey results and later eliminated? If the latter, which entries were kept for analysis (eg, the first entry or the most recent)?<br>Not applicable. "Each student received a unique transaction number along with the invitation email, which provided details on the estimated survey duration (20 to 30 minutes), voluntary participation, anonymity, data protection, and incentives (e.g., a chance to win VIP tickets for sporting events). Non-respondents received two reminders within a two-week period."	4, ll. 153-7
Analysis			
	Handling of incomplete questionnaires	Were only completed questionnaires analyzed? Were questionnaires which terminated early (where, for example, users did not go through all questionnaire pages) also analyzed?<br>"There was no completeness check, but incomplete surveys were also included."	4, ll. 161-2
	Questionnaires submitted with an atypical timestamp	Some investigators may measure the time people needed to fill in a questionnaire and exclude questionnaires that were submitted too soon. Specify the timeframe that was used as a cut-off point, and describe how this point was determined.<br>Not applicable	
	Statistical correction	Indicate whether any methods such as weighting of items or propensity scores have been used to adjust for the non-representative sample; if so, please describe the methods.<br>Not applicable	
